# Metastatic Chordoma: A Diagnostic Challenge on Fine Needle Aspiration

**DOI:** 10.1155/2016/2187290

**Published:** 2016-01-03

**Authors:** Ghassan Tranesh, Aziza Nassar

**Affiliations:** Department of Pathology, Mayo Clinic, 4500 San Pablo Road, Jacksonville, FL 32224, USA

## Abstract

Chordomas are primary low grade malignant tumors of bone that usually arise within both ends of axial skeleton. The Notochord is a midline, ectoderm-derived structure that defines the phylum of chordates. Chordomas may pose difficult diagnostic challenges when encountered in secondary locations, such as lungs or other parenchymatous organs. We report the cytologic findings of a metastatic chordoma sampled through CT-scan guided fine needle aspiration (FNA) of lower lobe lung nodule in a 54-year-old man diagnosed with recurrent chordoma involving the lumber spine and paraspinal region.

## 1. Introduction

Chordomas are primary low grade malignant tumors of bone that usually arise within both ends of axial skeleton. Chordoma has a phenotype that recapitulates notochord. The notochord is a midline, ectoderm-derived structure that defines the phylum of chordates [1]. In humans, the degeneration of the notochord begins during fetal development and is completed by the second decade of life [2]. Some controversy exists as to the origin of chordomas from these cells because chordomas arise from bone (clivus, sacrum, and coccyx), and not the intervertebral disks [3]. Chordoma represents a low grade malignant mesenchymal neoplasm with distinct clinical and pathologic features and histologic similarities to the notochord. It represents approximately 5% of malignant bone tumors. Distant metastases, most commonly to the lung, occur late in disease in approximately 30% of cases, but mortality is most often related to recurrence and progression with local effects [4].

## 2. Case Report

### 2.1. Subject

**Figure 1:**
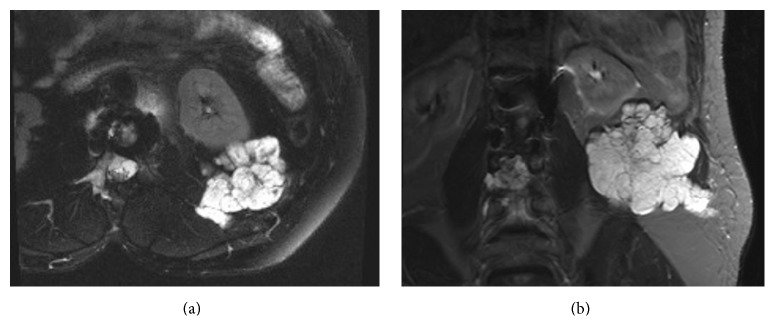
(a and b) Cross and Coronal sections MRI images show tumor recurrence with extension to psoas muscle.

**Figure 2:**
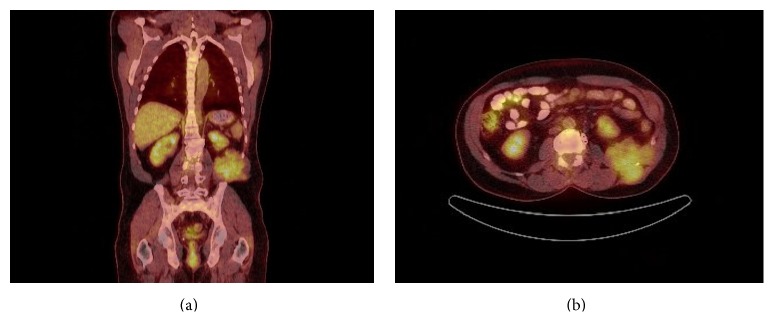
PET scan shows tumor recurrence.

**Figure 3:**
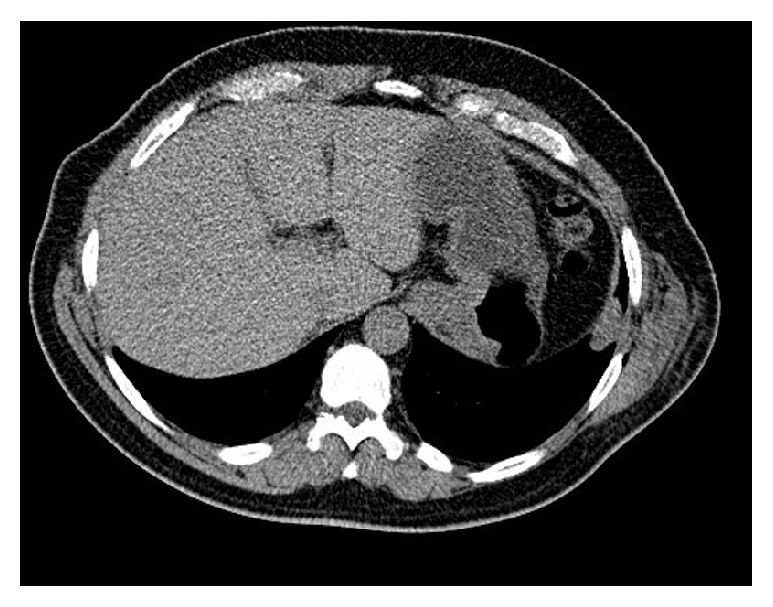
Chest CT-scan shows a 1.8 cm left lower lobe nodule.

**Figure 4:**
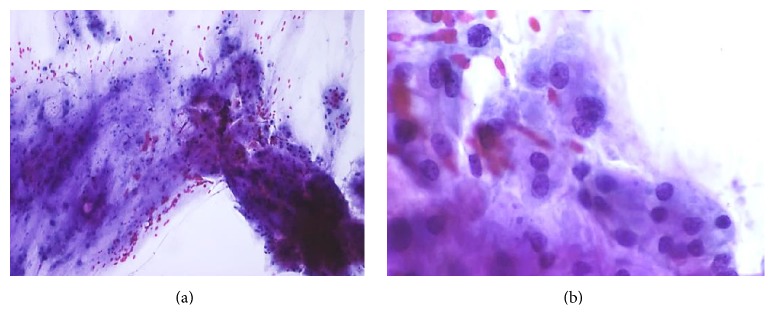
FNA Diff-Quik smears, low and higher power images show metastatic chordoma (fragments of fibrillary matrix material intimately admixed with epithelioid cells in a background of red blood cell).

The patient is a 54-year-old male with a history of lumbar and paraspinal chordoma. He presented initially with left gluteal, left thigh, and left groin pain that led to an MRI of the lumber spine that showed a lesion at L2 expanding to L4 with paraspinal involvement (Figures 1(a) and 1(b)). A CT-guided biopsy with appropriate immunohistochemical correlation confirmed the diagnosis of chordoma. The tumor was surgically excised. Few months later, the patient noticed a left flank lump and had another abdominal CT-scan and PET scan which showed tumor recurrence (Figures 2(a) and 2(b)). Chest CT-scan showed left lower lobe nodular density (Figure 3). Infectious or inflammatory etiologies were considered. A CT-guided fine needle aspiration (FNA) biopsy on the lung nodule showed metastatic chordoma (Figures 4(a) and 4(b)). Patient had laminectomy with debulking of the recurrent chordoma and postoperative radiotherapy. Patient began brachyury vaccine injections, which were well tolerated without significant reported toxicities.

### 2.2. Radiology

The initial abdominal MRI showed a lobulated and expansile multiseptated mass with markedly intense T2 signal. The mass is located anterior and lateral to the L3, L4, and L5 vertebral bodies, which is consistent with tumor or neoplastic lymphadenopathy. This mass lesion measured approximately 4.3 cm × 4.0 cm × 4.5 cm and abuts the lower abdominal aorta and the proximal left common iliac artery, without evidence for encasement. The tumor was surgically excised.

Few months following the surgical excision, abdominal MRI was performed and showed that residual/recurrent chordoma involving right posterior L2 elements with large right anterolateral epidural tumor extensions at L2 and L3 level has further progressed, now causing severe compromise of the thecal sac at L2-3 level. There was also interval increase in the size of the left retroperitoneal lymphadenopathy and the large left flank soft tissue metastasis. Tumor extension was also noted within the L2-L3 and L3-L4 neural foramina and right psoas muscle. There was no evidence of obvious intrathecal extension (Figures 1(a) and 1(b)).

Shortly after the MRI, patient had a full body PET scan which showed large low density hypermetabolic mass, SUV max 2.8, in the left flank involving the musculature of the left posterior abdominal wall and extending through the rib cage (Figures 2(a) and 2(b)).

Chest CT-scan showed borderline enlarged right hilar lymph node. There was no left hilar adenopathy with normal sized mediastinal and axillary lymph nodes. There was a soft tissue confluence in the left lower lobe measuring approximately 1.8 cm (Figure 3).

### 2.3. Cytologic Findings

Diff-Quik smears from the CT-guided FNA of the lung nodule were moderately cellular and showed multiple fragments of fibrillary matrix material intimately admixed with epithelioid cells in a background of red blood cells. The epithelioid cells were present in large branching three-dimensional groups associated with fibrillary matrix or as single cells and small loosely cohesive clusters. The fibrillary matrix appeared intensely magenta to purple in Diff-Quik-stained smears (Figures 4(a) and 4(b)). A cell block was prepared; however, there were no lesional cells for further ancillary studies. Previous resection slides were reviewed in conjunction with the current case. The histomorphologic features were identical. The previous resection immunostains showed the neoplastic cells to be strongly positive for cytokeratin AE1/AE3, Brachyury, and epithelial membrane antigen (EMA) and weakly positive for S100.

## 3. Discussion

Chordomas were first described by Lushka et al. in 1856 [5]. Chordomas occur mostly in the craniospinal axis and are thought to arise from notochordal remnants. Recent literature challenges this traditional concept suggesting that chordomas arise from preexisting benign notochordal cell tumors [6]. Chordomas usually present between the fourth and seventh decades with a male to female ratio of 2 : 1 [6]. Chordomas account for 5% of primary malignant bone tumors. Radiologically, chordomas are destructive, lytic masses which extend into the soft tissues, forming a sizable, well-defined mass which may show calcifications. Variety of genetic abnormalities can be identified in sporadic and familial tumors. Familial tumors (familial chordoma) have been associated with upregulation of T-brachyury, a nuclear transcription factor [7, 8]. Chordomas appear to be increased in incidence in patients with tuberous sclerosis [9]. Tumors in children usually arise in skull base and are often a part of tuberous sclerosis complex (TSC). Sacral chordomas have best prognosis and longest overall survival because they are most likely to be resected with negative margins. Local recurrences for sacrococcygeal tumors are common after incomplete excision with 5- and 10-year survival rates ranging from 60 to 95% and 40 to 60%, respectively [10]. The treatment of chordoma demands both aggressive resection and cautious attention to minimizing surgical tumor implantation.

The differential diagnosis of metastatic chordoma includes benign pulmonary hamartoma [11] which is composed of lobules of benign cartilage lacunes, myxoid stroma with adipose tissue. Also, pulmonary hamartomas are CK AE1/AE3 and brachyury negative [3, 12]. Metastatic chordoma can morphologically mimic mucinous carcinomas; besides, 3% of non-small cell pulmonary carcinomas may show brachyury immunostain positivity [13]. However, mucinous carcinomas usually show floating cohesive cell groups of uniform epithelial cells, often with lumen formation. Also, in this particular case, the clinical history and the radiological picture were not favoring mucinous carcinoma.

The other differential diagnosis is myxoid/round cell liposarcoma which is a spindle cell neoplasm, deeply located with plexiform vascular growth. However, it rarely occurs in the lung and in this case there were no lipoblasts or pleomorphic round cell component [12].

The other differential diagnosis is extraskeletal myxoid chondrosarcoma, which consists of multilobulated malignant cartilaginous growth. However, there were no anastomosing cellular cords or cartilaginous cells [13].

Brachyury is a transcription factor that is required for posterior mesoderm formation and differentiation as well as for the notochord development during embryogenesis. It is expressed in the neoplastic cells of chordoma, a malignant tumour deriving from notochordal remnants [14]. Brachyury immunostain is a synthetic peptide conjugated to KLH derived from within residues 250–350 of human brachyury [7].

Adjuvant therapy is controversial but known to contribute to long-term disease control and survival. Postoperative patient followup should continue long-term CT-scan surveillance; and any signs or symptoms consistent with possible local recurrence or metastasis should prompt a thorough workup. In the absence of symptoms, surveillance CT-scans can detect potential treatable recurrence. Recurrent disease can be treated aggressively with curative intent including surgery and adjuvant radiation therapy [15].

Brachyury vaccine has been designed to stimulate the human immune system to target chordoma. The vaccine is composed of heat-inactivated* S. cerevisiae* yeast that expresses human brachyury protein. This vaccine is the fifth of a novel class of yeast-based immunotherapeutics designed to stimulate immune responses to eliminate diseased cells. Patients who were vaccinated have shown a good tolerability profile in clinical programs to date. Expected adverse events from these products are limited to redness, swelling, and tenderness at the injection site, and mild, brief flu-like symptoms [16].
